# Weighted analysis of composite endpoints with simultaneous inference for flexible weight constraints

**DOI:** 10.1002/sim.7147

**Published:** 2016-10-26

**Authors:** Anh Nguyen Duc, Marcel Wolbers

**Affiliations:** ^1^ Oxford University Clinical Research Unit, Wellcome Trust Major Overseas ProgrammeHo Chi Minh CityVietnam; ^2^ Centre for Tropical Medicine, Nuffield Department of MedicineUniversity of OxfordOxfordU.K.

**Keywords:** composite endpoint, chi‐bar‐square distribution, conic constraints, multiplicity adjustment, simultaneous confidence intervals, weighted analyses

## Abstract

Composite endpoints are widely used as primary endpoints of randomized controlled trials across clinical disciplines. A common critique of the conventional analysis of composite endpoints is that all disease events are weighted equally, whereas their clinical relevance may differ substantially. We address this by introducing a framework for the weighted analysis of composite endpoints and interpretable test statistics, which are applicable to both binary and time‐to‐event data. To cope with the difficulty of selecting an exact set of weights, we propose a method for constructing simultaneous confidence intervals and tests that asymptotically preserve the family‐wise type I error in the strong sense across families of weights satisfying flexible inequality or order constraints based on the theory of ${\bar{\chi }}^{2}$‐distributions. We show that the method achieves the nominal simultaneous coverage rate with substantial efficiency gains over Scheffé's procedure in a simulation study and apply it to trials in cardiovascular disease and enteric fever. © 2016 The Authors. *Statistics in Medicine* Published by John Wiley & Sons Ltd.

## 1 Introduction

Composite endpoints combine the occurrence of multiple distinct disease events (also denoted as ‘component outcomes’ or ‘components’) to a single outcome measure and have been widely used as primary endpoints in randomized controlled clinical trials (RCTs) across clinical disciplines 1, 2, 3. Commonly, composite endpoints are analyzed as binary outcomes, that is, whether at least one of the components occurred during follow‐up or not, or as survival outcomes, that is, as the time to the first component outcome, and standard statistical tests are used for the comparison between treatment groups. The anticipated power gains from using a combined endpoint with a higher event rate compared with its components have been the main reason for their increasing popularity. However, composite endpoints have also been criticized for pooling components of varying clinical importance and for being driven by clinically less important component outcomes, which are observed more frequently and earlier 4, 5.

As a remedy, several authors stressed the importance of weighting the components to improve the clinical relevance and interpretation of composite endpoint analyses (e.g., 5, 6). Several approaches to the weighted analysis of composite endpoints have been proposed, but the resulting test statistics are somewhat difficult to interpret clinically 7, 8, 9. Moreover, despite attempts to quantify weights based on methods such as clinician‐investigator Delphi panels 10, discrete choice experiments among patients 11, or disability‐adjusted life years (DALY) lost 6, the choice of a single weight vector which is acceptable to all stakeholders is often difficult 4.

To circumvent these issues, we propose an alternative weighted test statistic with a straightforward clinical interpretation. As in other approaches, weights can be assigned to component outcomes, but we also allow assigning weights to more complex event types such as combinations or sequences of component outcomes as detailed in Section 2.2. While choosing a single quantitative weight vector might be problematic in many settings, weights should be non‐negative, and it is frequently possible to further rank components according to their relative importance. To exploit this, we introduce multiplicity adjustments for the proposed test statistic, which allow for simultaneous inference across a flexible set of weight vectors.

The remainder of the paper is structured as follows: Section 2 introduces a general framework for the weighted analysis of composite endpoint considering both binary and time‐to‐event endpoints. Section 3 proposes a strategy for simultaneous inference across multiple weighting schemes. A simulation study, which investigates the finite‐sample performance of the proposed multiplicity adjustment, is given in Section 4, followed by applications (Section 5) and concluding remarks (Section 6).

## 2 A framework for weighted analyses of binary and time–to–event composite endpoints

### 2.1 Notation and proposed test statistic

Assume that we want to compare two independent groups *A* and *B* in an RCT and that we are interested in the occurrence of *K* different clinical event types k=1,…,K over time with an associated predefined weights vector $w={\left(w_{1},\ldots ,w_{K}\right)}^{T}\in R^{K}$. In the context of composite endpoints, where all components are harmful, it is usually sensible to consider only non‐negative weights w⩾0. We first assume that all patients are followed‐up for the same predefined follow‐up period (0,*τ*] and propose the following weighted test statistic:

(1) $$\hat{\;\;T}\;(w,\tau )=\sum_{k=1}^{K}w_{k}\left({\hat{p}}_{A,k}(\tau )-{\hat{p}}_{B,k}(\tau ))\right)=w^{T}\hat{D}(\tau )$$

where ${\hat{p}}_{A,k}(\tau )$ and ${\hat{p}}_{B,k}(\tau )$ are the estimated absolute risks that an event type *k* occurs in the interval (0,*τ*] in groups *A* and *B*, respectively. For convenience, these quantities will be referred to as event type probabilities. Using $\hat{\;\;T}\;(w,\tau )$, one can then conduct Wald‐type confidence intervals for the population value T(w,τ) and associated significance test of the two‐sided (resp. one‐sided) null hypothesis $H_{0}:T(w,\tau )=0$ (resp. $H_{0}:T(w,\tau )⩽0$) against the alternative hypothesis $H_{A}:T(w,\tau )\neq 0$( $\text{resp.}\;H_{A:}T(w,\tau )>0$). Performing these tests is straightforward as long as $\hat{D}(\tau )$ follows an asymptotic normal distribution and a corresponding sample‐based estimator of its covariance matrix is available, which shall be discussed further in Section 2.3.

Importantly, the proposed test statistic is based on estimates of absolute risks of event types, which are the most relevant quantities for medical decision‐making. Moreover, it has a straightforward interpretation: If the weights are standardized to sum to one, $\hat{\;\;T}\;(w,\tau )$ is the weighted average of absolute risk differences for individual event types. Alternatively, if each weight *w*_*k*_ represents a certain type of ‘cost’ associated with event type *k*, then $\hat{\;\;T}\;(w,\tau )$ estimates the expected cost difference between the two interventions *A* and *B*.

The proposed test statistic ((1)) is sensible for binary data where only the status at time *τ* is known. It is also applicable to time‐to‐event data, if the planned follow‐up duration for all patients is the same, there is only mild right‐censoring, and the patient's status at time *τ* rather than the exact timing of events is of primary clinical interest. If the exact timing of events is available and subjects may be followed‐up for different durations, an integrated version of the statistic proposed in Equation  ((1)) may be more suitable and is defined as

(2) $$\hat{T^{\prime }}(w,\tau )=\sum_{k=1}^{K}w_{k}\overset{\tau }{\underset{0}{\int }}h(t)\left({\hat{p}}_{A,k}(t)-{\hat{p}}_{B,k}(t)\right)dt=w^{T}\hat{D^{\prime }}(\tau )$$

where *h*(·) is a non‐negative function of time and integration is up to a predefined maximum follow‐up time *τ*. Similar integrated test statistics have been proposed for comparing survival and cumulative incidence functions 12, 13, 14 and, in the survival setting, they have been shown to be a good competitor to the log‐rank test especially if proportional hazards does not hold 12, 15. The trivial function *h*(·) ≡ 1 is often used in practice, and for comparing survival functions, the test statistic can then be interpreted as a test of the difference in restricted means 15. However, *h*(·) could also be chosen to prioritize different time periods over others or to stabilize the estimate by giving lower weight to periods where censoring is high 12. Statistic ((2)) is an averaged version of ((1)) over time and expected to be more powerful for time‐to‐event data. However, it is also more complicated to interpret, requires the additional choice of the function *h*(·), and the covariance matrix of $\hat{D^{\prime }}(\tau )$ is more difficult to estimate as we shall discuss in Section 2.3.

### 2.2 Event type definition

If the number of components of a composite endpoint is large and subjects can experience more than one component outcome, many different combinations of component outcomes may be observed for each subject over time. In principle, each unique combination can constitute an event type in the terminology of the previous section (‘exhaustive’ setting), but there are also simpler settings that require the assignment of weights to a more manageable number of event types, which we call ‘competing risks’, ‘worst event’, and ‘marginal’ settings.

To simplify the discussion, we assume in this and the next section that the composite endpoint has only two component outcomes consisting of one fatal (*F*) and one non‐fatal (*N*) event, but the extension to more complex settings is straightforward. In this case, the exhaustive set of binary event types is *N* ^+^ *F* ^− ^, that is, having a non‐fatal but not a fatal event in the interval (0,*τ*],*N* ^−^ *F* ^+ ^,*N* ^+^ *F* ^+ ^, and *N* ^−^ *F* ^− ^. Note that the event type definition depends on the chosen time horizon (0,*τ*] and that their probabilities may not monotonically change over time, for example, the probability of *N* ^+^ *F* ^− ^ might initially increase but decrease again for larger values of *τ* when more patients also experience event *F*. Thus, it is important that the follow‐up period is chosen to be sufficiently long to allow for a clear clinical assessment of the merits of the two treatments with respect to their influence on all event types. This exhaustive setting partitions the set of all subjects into four mutually exclusive event type categories, and it appears sensible to prioritize event types that are severe implying that $w_{N^{+}F^{+}}⩾w_{N^{-}F^{+}}⩾w_{N^{+}F^{-}}⩾w_{N^{-}F^{-}}$. Usually, *N* ^−^ *F* ^− ^(i.e., no event) is excluded from the test statistic altogether (corresponding to $w_{N^{-}F^{-}}=0$), which has the additional benefit that the resulting covariance matrix of the test statistic $\hat{D}(\tau )$ is of full rank, a requirement for the multiplicity adjustment presented in Section 3.

An alternative setting with fewer event types, which we call the competing risks setting, is to focus on the type of the first event only: *N* ^+ ^, that is, experiencing the non‐fatal event as a first event in (0,*τ*],*N* ^−^ *F* ^+ ^, and *N* ^−^ *F* ^− ^. Similar to the exhaustive setting, all event types considered here are mutually exclusive. Indeed, the test statistic ((1)) associated with the competing risks setting is a special case of the test statistic for the exhaustive setting with the additional weight constraint $w_{N^{+}}=w_{N^{+}F^{-}}=w_{N^{+}F^{+}}$, which appears difficult to justify clinically. Of note, the conventional analysis of composite endpoints is a special case of the competing risks setting with equal weights for all event types.

Several authors have considered a priority ranking of different component outcomes and then focusing on the worst component outcome a patient experiences 16, 17, 18. A related idea is to assign weights to the patients worst event only, leading to the following event types: *F* ^+ ^ (death with or without a prior non‐fatal event), *N* ^+^ *F* ^− ^, and *N* ^−^ *F* ^− ^. Such a weighting scheme might be appropriate if the patient's worst component dominates the clinical assessment of the severity of their overall outcome or for weights based on considerations of DALY lost associated with different events 6.

The final setting that we consider is the marginal setting with the following event types: *N* ^+ ^ and *F* ^+ ^ (death with or without a prior non‐fatal event). The major difference between this setting and the previous ones is that the event types are not exclusive, that is, subjects may experience more than one event type. Of note, the marginal setting can also be viewed as a special case of the exhaustive setting with the additional weight constraint $w_{N^{+}F^{+}}=w_{N^{-}F^{+}}+w_{N^{+}F^{-}}$. Such an additivity constraint appears reasonable in many circumstances, for example, if the weights are based on considerations of financial costs associated with different component outcomes.

Of note, for non‐overlapping event type definitions (exhaustive, competing risks, and worst event), a trivial weight assignment of one to all event types implies a comparison of the overall risk of any event between the treatment groups for test statistic ((1)) and a comparison of the integrated survival functions of the time to the first event for test statistic ((2)). Thus, the conventional analysis of composite endpoints is a special case of our approach except that in the time‐to‐event setting, the log‐rank test is more frequently used in practice than the difference between the integrated survival functions. For the marginal event type definition, even trivial weights lead to a different comparison than the conventional analysis. However, the marginal event setting has the advantage that weighting schemes that assign a weight of 1 to one marginal event and weights of 0 to all others provide a comparison of component endpoints as a special case. The same is possible for the exhaustive setting (if weights of 1 are assigned to event types including the respective marginal event only) but not for the competing risks or worst event settings.

### 2.3 Distribution and power of the proposed test statistic

The simplest scenario under which $\hat{D}(\tau )$ follows an asymptotic normal distribution is when all subjects have complete follow‐up until time *τ*. For the exhaustive setting, the associated event type probabilities can be consistently estimated by the observed proportions, and an estimate $\hat{V}$ of the covariance matrix of $\hat{D}(\tau )$ can easily be derived from the underlying multinomial distributions. Consequently, the asymptotic variance of $\hat{\;\;T}\;(w,\tau )$ is $w^{T}\hat{V}w$.

The same derivation can also be applied to the competing risks and worst event settings. For the marginal setting, which has overlapping event types, the asymptotic covariance of $\hat{D}(\tau )$ can be derived from the corresponding exhaustive setting by noting that the required event type probabilities are linear transformations of the exhaustive ones. For example, for a fatal and a non‐fatal event, the corresponding linear transformation for the marginal setting is

(3) $$\left(\begin{array}{c} {\hat{p}}_{A,N^{+}}(\tau )\\ {\hat{p}}_{A,F^{+}}(\tau ) \end{array}\right)=\left(\begin{array}{ccc} 1 & 0 & 1\\ 0 & 1 & 1 \end{array}\right)\left(\begin{array}{c} {\hat{p}}_{A,N^{+}F^{-}}(\tau )\\ {\hat{p}}_{A,N^{-}F^{+}}(\tau )\\ {\hat{p}}_{A,N^{+}F^{+}}(\tau ) \end{array}\right)$$

For time‐to‐event data where exact event times are available but some subjects are right‐censored before time *τ*, inference for $\hat{D}(\tau )$ must be based on the underlying multistage model. The nonparametric Aalen–Johansen estimator provides consistent estimates of the event type probabilities even if the underlying multistage model is not Markov 19. Moreover, estimates and a corresponding Greenwood‐type estimate of the associated covariance matrix are readily available in standard software 20.

Of note, in the exhaustive setting, it is most convenient to derive these quantities based on a multistage model that is a directed tree with a single node for each event type. This is illustrated for an illness‐death model in Figure S1 of Appendix A. For the other settings, the corresponding quantities can be derived from the exhaustive setting via linear transformations as illustrated earlier for the competing risks setting. Alternatively, for the competing risks setting, the quantities can be derived based on a simplified multistage model, which includes only transitions from the initial state.

**Figure 1:**
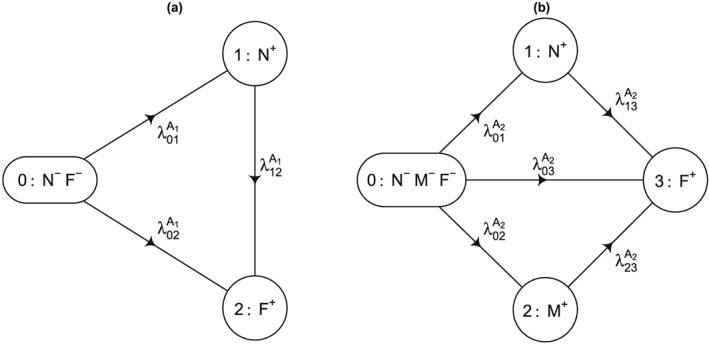
Multistage models for the simulation. All transition rates (per year of follow‐up) were assumed to be constant. (a) First series: $\lambda_{01}^{A_{1}}=0.05,\lambda_{02}^{A_{1}}=0.02$, and $\lambda_{12}^{A_{1}}=0.2$; (b) Second series: $\lambda_{01}^{A_{2}}=0.08,\lambda_{02}^{A_{2}}=0.1,\lambda_{03}^{A_{2}}=0.04,\lambda_{13}^{A_{2}}=0.2$, and $\lambda_{23}^{A_{2}}=0.3$.

The integrated test statistics $\hat{D^{\prime }}(\tau )$ can also be calculated from the Aalen–Johansen estimator by approximating the integral with a simple Riemann sum over a regular time grid from 0 to *τ* or via more complex numerical integration algorithms. While explicit estimators of its covariance matrix have been given in special cases including the survival and the competing risks setting 13, 14, the covariance is not readily available from standard software. As a pragmatic solution, we suggest estimating the covariance matrix as the sum of jackknife estimates of the covariance matrices of the integrated probabilities in both treatment arms. Alternatively, other resampling methods such as the bootstrap can be used 21.

The power of the proposed weighted test statistic ((1)) to detect difference between groups is illustrated in Appendix B of the Supporting Information for a simple example. It demonstrates that a weight vector with maximal power can be determined, which in most situations outperforms both the conventional analysis of the composite endpoint and the analysis of each individual component outcomes. However, importantly, weights should not primarily be chosen based on power considerations but on clinical grounds.

## 3 Simultaneous inference for the weighted analysis of composite endpoints

While non‐trivial weight vectors will often give more interpretable results than the conventional analysis, which implicitly selects equal weights for all first event types, the difficulty of choosing a single vector of quantitative weights acceptable to all stakeholders remains. However, as all components of a composite endpoint are usually harmful, weights should always be non‐negative, and it will often be possible to rank the event types in terms of their clinical importance or to define even more stringent linear equality or inequality constraints for the weights. Thus, it seems desirable to control multiplicity across all acceptable weight vectors satisfying such constraints and thus allowing different stakeholders to chose different weight vectors for their interpretation of the results without inflating the overall type I error. In this section, a flexible technique for simultaneous inference is introduced, which is based on the work by Shapiro 22. It generalized the classical method of Scheffé 23 for simultaneous inference across all possible contrasts to cone constraints.

By definition, a set C in $R^{K}$ is a cone if for all w∈C and for all r>0,rw∈C. For any chosen closed and convex cone C in $R^{K}$, Shapiro 22 suggested an approach for deriving simultaneous confidence intervals with a desired overall coverage probability for the quantities $w^{T}D$ with w∈C based on an observation $\hat{D}$ from a multivariate normal distribution N(D,V) with a known non‐singular covariance matrix *V*.

For most practical purposes including the ones in this paper, it is sufficient to consider cones C of the following form:

(4) $$C=\left\{w\in R^{K}:a_{i}^{T}w=0,i=1,\ldots ,s;a_{i}^{T}w⩾0,i=s+1,\ldots ,K\right\}$$

where the *K* × *K* matrix ${\left(a_{1}^{T},\ldots ,a_{K}^{T}\right)}^{T}$ is of full rank and at least one of the constraints is an inequality constraint.

For $Z(C,V):=\max_{w\in C}\frac{{\left(\hat{D}-D\right)}^{T}w}{{\left(w^{T}Vw\right)}^{1/2}}$, Shapiro 22 showed that ${\left[Z(C,V)\right]}^{2}$ has a chi‐bar squared ( ${\bar{\chi }}^{2}$) distribution 24, 25. By definition, a ${\bar{\chi }}^{2}$‐distribution is the weighted average of independent *χ* ^2^‐distributions, that is

(5) $$P(Z(C,V)⩾c)=\sum_{i=0}^{K}{\tilde{w}}_{i}(C,V^{-1})P\left(\chi_{i}^{2}⩾c\right)$$

where $\chi_{i}^{2}$ denotes a random variable following a central *χ* ^2^‐distribution with *i* degrees of freedom (with $\chi_{0}^{2}$ defined as a point mass at 0 by convention) and ${\tilde{w}}_{i}(C,V^{-1}),i=0,\ldots ,K$, are the associated weights, summing to one, which can be derived from the given cone C and the covariance matrix *V*. The exact derivation of the weights for cones defined according to Equation ((4)) is given in Section 5 of 25. This has been implemented for the special case $C=R_{+}^{K}$ in function wchibarsq of the R package varComp 26, and a general implementation is provided in Appendix E of the Supporting Information. Consequently, if $c_{1-\frac{\alpha }{2}}$ is the $\left(1-\frac{\alpha }{2}\right)$‐quantile of this ${\bar{\chi }}^{2}$‐distribution, simultaneous one‐sided lower $1-\frac{\alpha }{2}$ confidence intervals and two‐sided 1 − *α* confidence intervals for all $w^{T}D$ with w∈C are given by

(6) $$\begin{array}{cc} CI_{L}\left(w,V,c_{1-\frac{\alpha }{2}}\right) & =\left[w^{T}\hat{D}-c_{1-\frac{\alpha }{2}}^{1/2}{\left(w^{T}Vw\right)}^{1/2},+\infty \right)\text{and}\\ CI\left(w,V,c_{1-\frac{\alpha }{2}}\right) & =\left[w^{T}\hat{D}-c_{1-\frac{\alpha }{2}}^{1/2}{\left(w^{T}Vw\right)}^{1/2},w^{T}\hat{D}+c_{1-\frac{\alpha }{2}}^{1/2}{\left(w^{T}Vw\right)}^{1/2}\right]. \end{array}$$

In Appendix C of the Supporting Information, it is shown that Shapiro's result also applies to the asymptotic setting. Specifically, if $\hat{D}(\tau )$ follows an asymptotic normal distribution, and a consistent estimator $\hat{V}$ of its covariance matrix is available, then asymptotically valid simultaneous confidence intervals for $T(w,\tau )=w^{T}D(\tau )$ for all weight vectors w∈C can be obtained via formula ((6)) by using $\hat{V}$ instead of *V* in that formula and in the derivation of the required ${\bar{\chi }}^{2}$‐weights ${\tilde{w}}_{i}$.

Using the duality between hypothesis testing and confidence intervals, these simultaneous confidence intervals can also be used to derive associated tests, which control the family‐wise type I error in the strong sense across the two‐sided or one‐sided null hypotheses $H_{0}:w^{T}\hat{D}(\tau )=0$ or $H_{0}:w^{T}\hat{D}(\tau )⩽0$, respectively, with weights fulfilling the desired constraints. Specifically, for any weight vector fulfilling the constraints, the associated hypothesis test is rejected if zero is not contained in the associated simultaneous confidence interval.

## 4 Simulation studies

The finite‐sample performance of the multiplicity adjustment discussed in Section 3, hereafter called ${\bar{\chi }}^{2}$‐method, was evaluated in a simulation study and compared with an asymptotic version of Scheffé's method 23, which guarantees simultaneous control across all weight vectors without any constraints, and unadjusted confidence intervals.

### 4.1 Scenarios and assessment methods

Two series of simulation scenarios were investigated, both imitating RCTs with a 1:1 randomization of an intervention versus control and a maximum follow‐up duration of *τ* = 5 (years). For the first series, data in the control arm *A*_1_ were simulated based on an illness‐death model with a less severe (non‐fatal) state *N*, an absorbing (fatal) state *F*, and constant transition hazards as per Figure 1(a). For the second series, data in the control arm *A*_2_ were simulated according to a more general multistage model with one fatal (absorbing) state *F* and two transient states *N* and *M* as per Figure 1(b), where event *M* was assumed to be more severe than *N*. For each series, we considered three treatment effect scenarios: *B*_*j*_,*C*_*j*_, and *D*_*j*_(*j* = 1,2) simulated according to the same respective multistage models. Intervention *B*_*j*_ was assumed to have no effect, intervention *C*_*j*_ was assumed to reduce all transition hazards by 25*%*, and intervention *D*_*j*_ was assumed to only affect the transition hazard $\lambda_{01}^{A_{j}}$ and reduce it by 25*%*. This leads to six scenarios across the two simulation series.

Besides scenarios with complete follow‐up, that is, only administrative right‐censoring at time *τ* = 5, scenarios with both administrative right‐censoring and independent right‐censoring following an exponential distribution with rate *λ* = 0.05 were investigated. The resulting proportion of right‐censored observations before time *τ* ranged from 17*%* to 19*%* across all settings. The resulting event probabilities for all settings are shown in Tables S2 and S3 in Appendix D of the Supporting Information. Finally, we varied the sample size between 100 and 500 per arm, which implied a total of 24 scenarios.

In all scenarios, evaluations were based on test statistic ((1)). Both exhaustive and marginal event type definitions were investigated with either only non‐negativity constraints for all weights or an order constraints that prioritized event types comprising more severe outcomes (e.g., for the first series, the order constraints were $w_{F^{+}N^{+}}⩾w_{F^{+}N^{-}}⩾w_{F^{-}N^{+}}$ for the exhaustive and $w_{F^{+}}⩾w_{N^{+}}$ for the marginal setting).

The number of simulations per scenario was 10,000 and the primary evaluation criterion was the simultaneous Monte Carlo coverage probability of nominal two‐sided 95*%* confidence intervals across weight vectors satisfying the respective constraints. As there are an infinite number of weight vectors satisfying the constraints, simultaneous coverage was approximated by evaluating it on a grid of uniformly spaced points across the intersection of the cone with the hyperplane defined by the constraint that all weights sum to one. For intersections of dimensions 1, 2, and 4, the corresponding grids had approximately 1000, 3000, and 10,000 points, respectively.

All examined confidence intervals have the form

(7) $$\left[w^{T}\hat{D}(\tau )-{\left(\eta w^{T}\hat{V}w\right)}^{1/2},w^{T}\hat{D}(\tau )+{\left(\eta w^{T}\hat{V}w\right)}^{1/2}\right]$$

where *η* is either *c*_0.975_, that is, the 97.5*%* quantile of the ${\bar{\chi }}^{2}$‐distribution corresponding to the approximate covariance matrix $\hat{V}$ and the cone constraint for the ${\bar{\chi }}^{2}$‐method, or *s*_0.95_, that is, the 95*%* quantile of the *χ* ^2^‐distribution with degrees of freedom equal to the number of event types for the asymptotic Scheffé's method, or the 95*%* quantile of the *χ* ^2^‐distribution with one degree of freedom, that is, 1.96^2^, for the unadjusted method.

To study the cost of controlling simultaneous coverage to the unadjusted method, we also calculated the relative efficiency of the ${\bar{\chi }}^{2}$‐ method and Scheffé method defined as $c_{0.975}^{1/2}/1.96$ and $s_{0.95}^{1/2}/1.96$, respectively.

### 4.2 Results

Simulation results for the exhaustive and marginal settings for the 24 scenarios are displayed in Tables 1 and 2, respectively. Coverage for the ${\bar{\chi }}^{2}$‐method was close to the nominal 95*%* and between 94*%* and 96.1*%* across all scenarios including those involving right‐censoring. For *n* = 500, coverage more than two Monte Carlo standard errors below 95*%*, that is, coverage below 94.6*%*, occurred for only 2/48 (both being 94.5*%*) reported coverage probabilities. For *n* = 100, mild under‐coverage occurred more frequently, and the lowest observed coverage of 94.0*%* was observed in the highest dimensional setting with five exhaustive event types. As expected, Scheffé's method yielded coverage beyond the nominal level, whereas the unadjusted confidence intervals had substantial simultaneous under‐coverage.

**Table 1:** Simulation results for ‘exhaustive’ settings.

Groups	*n*	MC relative efficiency			MC simultaneous coverage *%* ^*@*^					
		${\bar{\chi }}^{2*}$			${\bar{\chi }}^{2*}$		Scheffé^*$*^		Unadjusted^*#*^	
Weight constraints		$R_{+}^{p}$	⩽	Scheffé^*$*^	$R_{+}^{p}$	⩽	$R_{+}^{p}$	⩽	$R_{+}^{p}$	⩽
No right‐censoring before *τ*										
*A*_1_&*B*_1_	100	1.36	1.21	1.43	94.7	94.4	96.2	98.0	76.1	86.3
	500	1.36	1.21	1.43	95.1	95.1	96.4	98.5	77.4	87.4
*A*_1_&*C*_1_	100	1.35	1.22	1.43	94.5	94.8	96.2	98.0	76.0	86.5
	500	1.36	1.22	1.43	95.1	94.9	96.5	98.1	77.3	87.1
*A*_1_&*D*_1_	100	1.36	1.21	1.43	95.0	94.8	96.5	98.5	76.3	86.7
	500	1.36	1.21	1.43	94.8	94.8	96.4	98.3	77.0	86.5
*A*_2_&*B*_2_	100	1.63	1.31	1.70	94.4	94.7	95.9	99.2	47.1	80.9
	500	1.63	1.31	1.70	95.1	95.2	96.6	99.4	49.3	81.3
*A*_2_&*C*_2_	100	1.62	1.31	1.70	94.0	94.9	95.9	99.5	48.3	81.1
	500	1.62	1.31	1.70	94.9	94.9	96.6	99.3	50.3	81.6
*A*_2_&*D*_2_	100	1.63	1.30	1.70	94.5	94.6	96.3	99.3	48.1	81.9
	500	1.63	1.30	1.70	94.9	94.9	96.5	99.4	49.2	81.8
Right‐censoring before *τ*										
*A*_1_&*B*_1_	100	1.36	1.21	1.43	94.6	94.4	96.3	98.3	76.0	86.4
	500	1.36	1.22	1.43	94.8	95.1	96.5	98.2	76.9	87.0
*A*_1_&*C*_1_	100	1.36	1.22	1.43	94.7	94.8	96.3	98.0	75.8	86.2
	500	1.36	1.22	1.43	94.7	94.5	96.3	98.2	77.4	86.6
*A*_1_&*D*_1_	100	1.36	1.22	1.43	94.5	94.5	96.2	98.1	76.1	86.4
	500	1.36	1.22	1.43	95.2	95.0	96.7	98.4	77.0	86.9
*A*_2_&*B*_2_	100	1.63	1.31	1.70	94.4	94.8	95.9	99.3	47.4	81.4
	500	1.63	1.31	1.70	95.2	95.1	96.6	99.4	48.5	81.3
*A*_2_&*C*_2_	100	1.62	1.31	1.70	94.0	94.6	95.9	99.2	47.1	80.6
	500	1.62	1.31	1.70	95.1	95.3	96.8	99.5	51.2	81.8
*A*_2_&*D*_2_	100	1.63	1.30	1.70	94.4	94.2	96.0	99.3	47.4	80.9
	500	1.63	1.30	1.70	95.0	95.0	96.5	99.5	49.9	81.8

*, Resulting from simultaneous confidence interval based on ${\bar{\chi }}^{2}$‐method.

$, Resulting from simultaneous confidence interval based on Scheffé's method.

# Resulting from unadjusted simultaneous confidence interval.

$R_{+}^{p}$, Non‐negativity constraint, *p* = 3 for *A*_1_,*B*_1_,*C*_1_ & *D*_1_ and *p* = 5 for *A*_2_,*B*_2_,*C*_2_ & *D*_2_

⩽, Order constraint.

@ Monte Carlo standard error ≈0.2*%*.

**Table 2:** Simulation results for ‘marginal’ settings.

Groups	*n*	MC relative efficiency			MC simultaneous coverage *%* ^*@*^					
		${\bar{\chi }}^{2*}$			${\bar{\chi }}^{2*}$		Scheffé^*$*^		Unadjusted^*#*^	
Weight constraints		$R_{+}^{p}$	⩽	Scheffé^*$*^	$R_{+}^{p}$	⩽	$R_{+}^{p}$	⩽	$R_{+}^{p}$	⩽
No right‐censoring before *τ*										
*A*_1_&*B*_1_	100	1.17	1.11	1.25	94.2	94.2	96.0	97.1	88.4	91.2
	500	1.17	1.11	1.25	94.8	95.0	96.4	97.5	89.3	91.9
*A*_1_&*C*_1_	100	1.17	1.11	1.25	94.2	94.6	95.9	97.1	88.5	91.3
	500	1.17	1.11	1.25	94.9	94.7	96.3	97.3	88.8	91.5
*A*_1_&*D*_1_	100	1.17	1.11	1.25	94.7	95.0	96.6	97.7	88.4	91.7
	500	1.17	1.11	1.25	94.5	94.7	96.4	97.4	88.8	91.6
*A*_2_&*B*_2_	100	1.34	1.20	1.43	94.4	95.6	96.5	98.6	77.9	89.4
	500	1.34	1.20	1.43	95.1	95.9	96.9	98.7	78.2	89.5
*A*_2_&*C*_2_	100	1.34	1.20	1.43	94.6	95.9	96.6	98.7	78.5	90.0
	500	1.34	1.20	1.43	95.0	95.8	96.7	98.5	78.7	89.8
*A*_2_&*D*_2_	100	1.34	1.20	1.43	94.5	95.6	96.4	98.5	78.3	89.7
	500	1.34	1.20	1.43	94.8	95.8	96.6	98.6	78.6	89.9
Right‐censoring before *τ*										
*A*_1_&*B*_1_	100	1.17	1.11	1.25	94.3	94.4	96.1	97.2	88.1	90.9
	500	1.17	1.11	1.25	94.8	94.9	96.6	97.5	89.1	91.6
*A*_1_&*C*_1_	100	1.18	1.11	1.25	94.7	94.6	96.3	97.5	88.2	91.3
	500	1.18	1.11	1.25	94.7	94.6	96.2	97.3	88.6	91.4
*A*_1_&*D*_1_	100	1.17	1.11	1.25	94.4	94.7	96.1	97.3	88.5	91.5
	500	1.17	1.11	1.25	95.2	95.0	96.7	97.6	89.0	91.6
*A*_2_&*B*_2_	100	1.34	1.21	1.43	94.6	95.9	96.3	98.6	78.3	89.6
	500	1.34	1.21	1.43	95.2	95.9	96.7	98.7	78.9	89.8
*A*_2_&*C*_2_	100	1.34	1.20	1.43	94.4	95.6	96.4	98.6	77.5	89.1
	500	1.34	1.20	1.43	94.9	96.1	96.8	98.8	79.4	90.4
*A*_2_&*D*_2_	100	1.34	1.20	1.43	94.7	95.4	96.4	98.6	78.1	89.7
	500	1.34	1.20	1.43	95.0	96.1	96.8	98.8	78.7	90.0

* Resulting from simultaneous confidence interval based on ${\bar{\chi }}^{2}$ method.

$, Resulting from simultaneous confidence interval based on Scheffé's method.

#, Resulting from unadjusted simultaneous confidence interval.

$R_{+}^{p}$, Non‐negativity constraint, *p* = 2 for *A*_1_,*B*_1_,*C*_1_ & *D*_1_ and *p* = 3 for *A*_2_,*B*_2_,*C*_2_ & *D*_2_

⩽, Order constraint.

@, Monte Carlo standard error ≈0.2*%*.

Monte Carlo relative efficiency followed the anticipated pattern that relative to the unadjusted method, the ${\bar{\chi }}^{2}$‐method is more efficient than Scheffé's method, especially under the more restrictive constraint as Scheffé's method disregards these weight restrictions. Compared with the unadjusted confidence intervals, confidence intervals for the ${\bar{\chi }}^{2}$‐method under an order constraint were approximately 21*%* to 31*%* wider for the exhaustive and 11*%* to 21*%* wider for the marginal setting. In contrast, Scheffé's method led to increases by 43*%* to 70*%* and 25*%* to 43*%*, respectively. The gain by using the ${\bar{\chi }}^{2}$‐method over Scheffé's method was more substantial as the dimension of the weight vector increased.

## 5 Applications

### 5.1 Design consideration for a cardiovascular trial

In this case study, we consider the design of a hypothetical RCT in cardiology based on the work of Hong *et al.* 6, who suggested assigning weights based on standardized DALY to the following three common outcomes in vascular prevention trials: non‐fatal myocardial infarction (*M* *I* ^+ ^), non‐fatal stroke (*S* *T* ^+ ^), and vascular death (*D* *E* ^+ ^). The corresponding reported weight vectors (DALYs) for ages 50, 60, and 70 were *w*_50_ = (6.73,10.49,16.79),*w*_60_ = (5.14,7.63,11.59), and *w*_70_ = (3.85,5.06,7.24) (see Table 2 in 6). Hong *et al.* 6 did not consider the setting of multiple events per patients, and we assume here that their weights refer to a ‘worst event’ setting.

**Figure 2:**
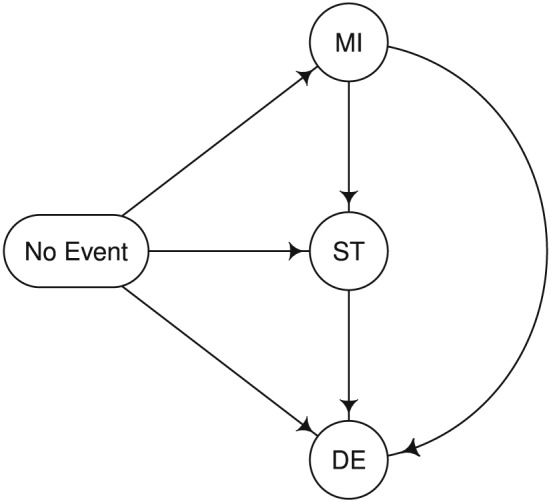
Multistage model for the cardiovascular trial example. The transition rates (per year of follow‐up) in the control and intervention arm, respectively, were assumed to be as follows: *λ*_No Event→MI_ = 0.04 vs. 0.03, *λ*_No Event→ST_ = 0.06 vs. 0.04, *λ*_No Event→DE_ = 0.015 vs. 0.01, *λ*_MI→ST_ = 0.12 vs. 0.08, *λ*_MI→DE_ = 0.03 vs. 0.02, and *λ*_ST→DE_ = 0.03 vs. 0.02.

For the design of a 1:1 RCT in a stroke‐prone population of control versus intervention, we assumed that data follow the multistage model displayed in Figure 2. This model assumes constant initial cause‐specific hazards of 0.04 and 0.03 MI events/year, 0.06 and 0.04 ST events/year, and 0.015 and 0.01 DE events/year in the control and intervention groups, respectively, and that the respective transition rates double after an initial event. Note that we did not model transitions from ST to MI for simplicity and because we focused on the subjects' worst event type. During a follow‐up period of 3 years, this scenario implies that 29.2*%* of participants experience an event in the control group (probability of worst events: 8.1*%* for MI, 16.1*%* for ST, and 5.0*%* for DE) compared with 21.3*%* (probability of worst events: 6.9*%* for MI, 11.3*%* for ST, and 3.2*%* for DE) in the intervention arm. Moreover, we assumed that non‐administrative censoring during the 3‐year follow‐up period followed an exponential distribution with a rate of 0.05 censoring events/year.

Based on this scenario, sample size calculations were performed using simulation. We first focused on test statistic ((1)) and estimates of event probabilities by 3 years based on the Nelson–Aalen estimator to account for censoring. For a conventional analysis of the composite endpoint, which compares the overall risk of any event, a sample size of 700 subjects per group would be sufficient to detect a difference between the two groups with 90*%* power at the two‐sided 5*%* significance level. A compatible analysis that gives the same weight to all worst events for the main analysis but allows for multiplicity‐corrected simultaneous inference across all ordered non‐negative weights (respecting the ordering $w_{DE}⩾w_{ST}⩾w_{MI}$) would require a sample size of 910 subjects per group, that is, a 30*%* increase in sample size. Such an increase may seem considerable. However, note that a Bonferroni correction for the analysis of the composite endpoint and all three component events would require a sample size increase of 36*%* while only allowing for multiple comparisons across fewer and arguably less clinically relevant null hypotheses. Finally, we calculated the required sample for 90*%* power to detect an intervention effect simultaneously across all weights in the convex cone spanned by the age‐dependent DALY weight vectors *w*_50_,*w*_60_, and *w*_70_ given earlier using simulation. The resulting sample size was 685 subjects per group. Interestingly, this is a smaller sample size than for the conventional analysis, which may be due to two factors. First, the weight vectors *w*_50_,*w*_60_, and *w*_70_ all give lowest weight to non‐fatal *MI* whose assumed intervention effect was smallest. Second, despite being quantitatively different, the weight vectors span a ‘narrow’ cone, hence requiring only minimal multiplicity adjustment.

In a second step, we focused on analyses based on the integrated test statistic ((2)) with a weighting function *h*(·) ≡ 1 over time. For this setting, a sample size calculation based on ((2)) with equal weights for all event types and no multiplicity adjustment lead to a sample size of 755 subjects per group for 90*%* power. Thus, in this setting, with proportional hazards of the time to the first event, the simpler analysis based on statistic ((1)) would be preferred to the integrated test statistic ((2)) as the resulting sample size is lower and interpretation is easier. As a comparison, a conventional log‐rank test comparing the time to the first event between the two groups would require a 9*%* lower sample size of 680 subjects per group. Finally, the required sample size for 90*%* power to detect an intervention effect simultaneously across all weights in the convex cone spanned by the age‐dependent DALY weight vectors based on statistic ((2)) was 740 subjects per group, that is, again a slightly lower sample size than for the unweighted analysis without multiplicity adjustment.

### 5.2 Weighted composite endpoint analysis in a trial of enteric fever

We applied the ${\bar{\chi }}^{2}$‐method to data from a typhoid trial studying the efficacy of two antibiotic treatments for uncomplicated enteric fever: Gatifloxacin and Cefiximie 27. The outcome of interest here is the composite endpoint of overall treatment failure, which was a secondary endpoint in the trial. Overall treatment failure was defined as acute treatment failure (severe complications, fever or other persistent symptoms for more than 7 days, or requirement for rescue treatment), death, or relapse (fever with a positive blood culture within a month of completing treatment). Only one death occurred and it was pooled with acute treatment failure for the sake of this example. By definition, subjects with an acute treatment failure were not evaluated for relapse, hence the event types are exclusive.

**Figure 3:**
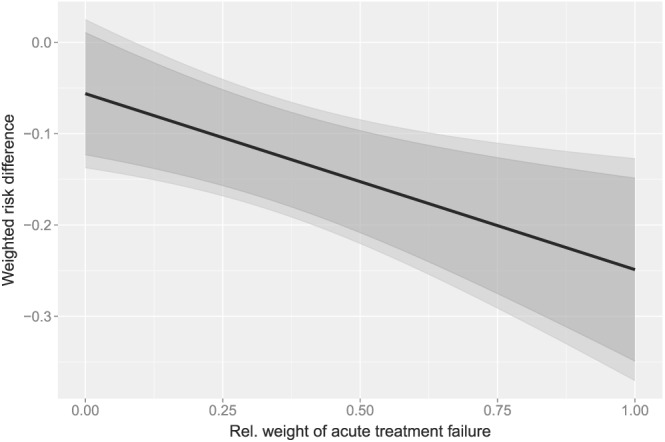
Weighted risk difference depending on the relative weight of ‘acute treatment failure or death’. Black line, weighted risk difference; dark gray, unadjusted 95*%* confidence intervals; light gray, simultaneous 95*%* confidence intervals.

The observed frequencies of ‘acute treatment failure or death’ and relapse in patients with culture‐confirmed typhoid were 1/92 and 2/92 in the Gatifloxacine arm and 20/77 and 6/77 in the Cefixime arm 27. As it is unclear which of the two event types is more clinically relevant in general, we did not impose an order constraint but instead adjusted for multiplicity taking into account only the non‐negativity constraint. Figure 3 shows the weighted test statistic $\hat{\;\;T}\;(w,\tau )$ and associated 95*%* confidence intervals (with and without multiplicity adjustment) depending on the weight assigned to ‘acute treatment failure or death’ and assuming that the two weights are standardized to sum up to 1.

According to Figure 3, across all weight choices, the associated 95*%* confidence intervals given by the ${\bar{\chi }}^{2}$‐ method are marginally wider than the unadjusted ones. Moreover, the simultaneous 95*%* confidence intervals based on the ${\bar{\chi }}^{2}$‐ method demonstrate a significant difference in the weighted risk differences if the relative weight of acute treatment failure or death is greater than 10*%*. As a relative weight below 10*%* for this component outcome seems unreasonable, the trial demonstrates superiority of Gatifloxacin over Cefixime with respect to the combined outcome simultaneously across all ‘clinically reasonable’ weights.

## 6 Discussion

We developed a new approach to the weighted analysis of composite endpoints applicable to both binary and time‐to‐event data. The proposed test statistic is a weighted absolute risk difference of component outcomes, which is both interpretable and clinically relevant. In practice, the choice of an exact set of weight vectors will often need to be based on a panel decision or health economics consideration 6, 10, 11, but we believe that a weighted analysis based on a predefined non‐trivial weight vector will usually lead to more clinically relevant and interpretable results than the conventional analysis, which implicitly assigns equal weights to all first‐event types and no weight to subsequent events. In some instances, such as in our cardiovascular design example, an adequately powered weighted analysis requires fewer subjects than the conventional analysis. However, frequently, the absolute risk decrease associated with an intervention will be smallest for the least frequent and most severe component endpoint mandating an increased sample size for the weighted analysis.

To account for the difficulties in choosing quantitative weights, we further proposed to use the ${\bar{\chi }}^{2}$‐method of 22 for simultaneous inference across flexible families of weight vectors satisfying a cone constraint. To our knowledge, this work is the first substantive application of Shapiro's method to a practical problem where constraints naturally arise. We extended this method to the asymptotic setting and demonstrated that it performs well for realistic sample sizes in a series of simulation studies. The proposed method allows to handle a flexible class of weight constraints that can be expressed as a system of linear equality and inequality constraints, which is general enough for most practical purposes.

In practice, the proposed method can be applied in two settings. First, it can supplement a conventional primary analysis with equal weights for all component outcomes by allowing for the additional exploration of non‐trivial and perhaps more clinically relevant weights without inflating the type I error. By controlling for multiplicity only across contrasts of clinical relevance, substantial efficacy gains are possible compared with Scheffé's method, which controls multiplicity across all contrasts. Second, a weighted analysis with weights and no multiplicity adjustment could be the primary analysis. Clearly, for a valid analysis, these weights need to be predefined at the planning stage. Alternatively, if precise weights are not available but a consensus could be found by a panel that all acceptable weight vectors are constrained to lie within a narrow cone, a multiplicity adjustment would be expected to require only a marginal increase in sample size as shown in our cardiovascular trial example.

Traditional methods for multiplicity adjustments of composite endpoints in RCTs focus on nonparametric simultaneous inference for the conventional analysis of the composite endpoint and its component outcomes, that is, a finite number of null hypotheses, and use multi‐step closed testing procedures to improve power 28, 29. In contrast, the proposed method is a single‐step parametric multiplicity adjustment across an infinite number of weights vectors satisfying flexible constraints. A major advantage of our approach is that weighted comparisons allow to explicitly trade off the importance of different components against each other for clinical decision‐making. Moreover, the method allows to perform both hypothesis tests and to calculate associated simultaneous confidence intervals, whereas the creation of compatible confidence intervals is difficult for multi‐step closed testing procedures 29. Of note, the current work relies on the accuracy of a parametric multivariate normal approximation to the Wald‐type test statistics. It is well known that Wald‐type confidence intervals are unreliable for low‐event probabilities and sample sizes even in simpler settings without multiplicity adjustment 30, and this may explain the observed mild under‐coverage for the lower sample size in our simulation study. Extending the current approach to likelihood‐ratio‐ or score‐based test statistics could be a potential area for future research.

Our approach focuses on absolute risks of event types. Absolute risks directly translate to expected event numbers in a population receiving a treatment and are the most relevant quantities for clinical decision‐making 31. By including appropriate weights, which allow to trade‐off the consequences of different event types against each other, our test statistic provides an overall assessment regarding which treatment has a more favorable effect on the overall frequency and composition of event types. As such, our approach answers a decision‐theoretic rather than an etiological question. Of note, absolute risks depend on the follow‐up horizon, which must be carefully chosen to allow for a reliable assessment of the effect of the interventions on the outcome. To answer etiological questions, regression models of the cause‐specific hazards of the underlying multistage model might be more appropriate, and it is well known that in the presence of competing risks, the effect of treatment on the absolute risk and the cause‐specific hazards might differ 32. As an example of the differences in the interpretation between models for absolute risks and cause‐specific hazards, assume that we are interested in the effect of a drug on a non‐fatal and a fatal event. Cause‐specific hazards models might reveal that the drug reduces the cause‐specific hazard of the fatal event but that it does not affect the rate of the non‐fatal events. On the absolute risk scale, this would imply a reduction in the overall absolute risk of any event as well as the risk of the fatal event but also an increase in the risk of the non‐fatal event because more subjects remain at risk to experience this event. As the weighted analysis essentially reduces to the conventional analysis for equal weights, our test statistic would also favor the drug if the non‐fatal event was assigned an equal or lower weight than the fatal event, which would usually be sensible. However, if the non‐fatal event was extremely severe and costly and thus assigned a larger weight than the fatal event, our weighted test statistic might not favor the drug.

Several extensions of our approach are possible. For example, there is no technical barrier that prevents using the developed framework for the joint analysis of beneficial and harmful outcomes by using weights of opposite signs. More generally, the proposed approach can be applied to the joint analysis of any set of multiple endpoints and arbitrary test statistics as long as they follow a joint asymptotic normal distribution. Another possible extension of the proposed approach is to consider test statistics that adjust for covariates other than treatment assignment including stratified versions of the proposed statistic.

## 7 Software

An R demonstration of the proposed method is provided in Appendix E of the Supporting Information.

## Supporting information

supporting info item
