# Supplementary material for: Weighted analysis of composite endpoints with simultaneous inference for flexible weight constraints
Source: Stat Med. 2016 Oct 26;36(3):442–54. doi: 10.1002/sim.7147 (PMC5217097; doi:10.1002/sim.7147)
Supplement: Supplementary file 1 — supporting info item [file SIM-36-442-s001.zip › weighted_compEP_supplements_rev.pdf]

Supplementary Material

Statistics in Medicine

(www.interscience.wiley.com)

Supplementary material for Weighted analysis of composite endpoints with simultaneous inference for flexible weight constraints

Anh Nguyen Duc[a] and Marcel Wolbers[a,b]*

A. The illness-death model as a directed tree

Figure 1 shows how an illness death model can be rewritten as a directed tree with a single node for each event type.

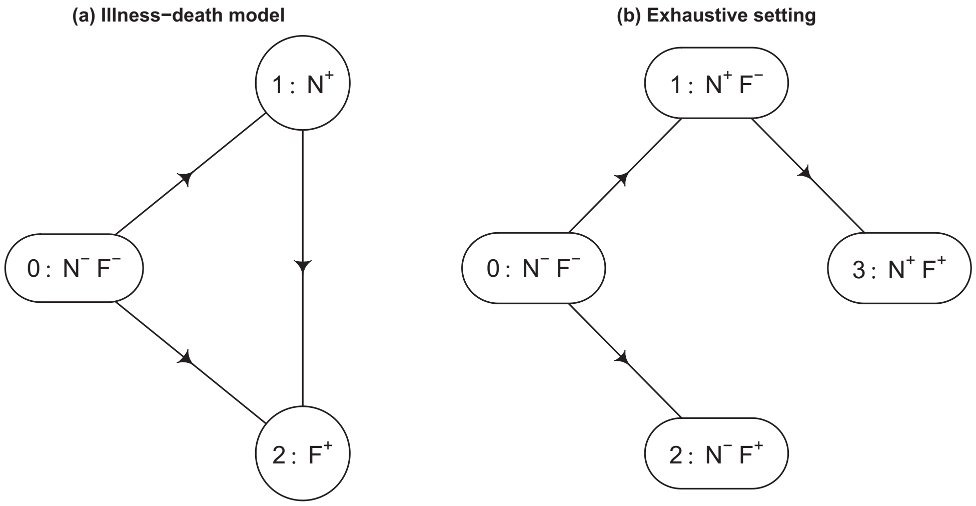

Figure 1. Conventional illness-death model and an equivalent multistage model with a distinct node for each "exhaustive" event type.

[a]*Oxford University Clinical Research Unit, Wellcome Trust Major Overseas Programme, Ho Chi Minh City, Viet Nam.*
[b]*Centre for Tropical Medicine, Nuffield Department of Medicine, University of Oxford, Oxford, UK.*
∗Correspondence to: Marcel Wolbers. E-mail: mwolbers@oucru.org

B. Power of the proposed test statistic – an illustration

The power of the proposed weighted test statistic (1) was investigated for a simple situation of two exclusive component outcomes and a control treatment and three different hypothetical interventions as given in Table 1. All three interventions reduce the absolute risk of the composite endpoint from 45% to 35% and according to a standard sample size calculation a sample size of $n = 503$ subjects per group would be required to detect such an effect with 90% power at the two-sided 5% significance level in a 1:1 randomized trial. Figure 2 displays the power of the suggested weighted comparisons for the same sample size depending on the chosen weights.

Table 1. Probability of a composite endpoint and its components by treatment arm.

	Component 1	Component 2	Composite endpoint
Control	15%	30%	45%
Intervention 1	10%	25%	35%
Intervention 2	8%	27%	35%
Intervention 3	14%	21%	35%

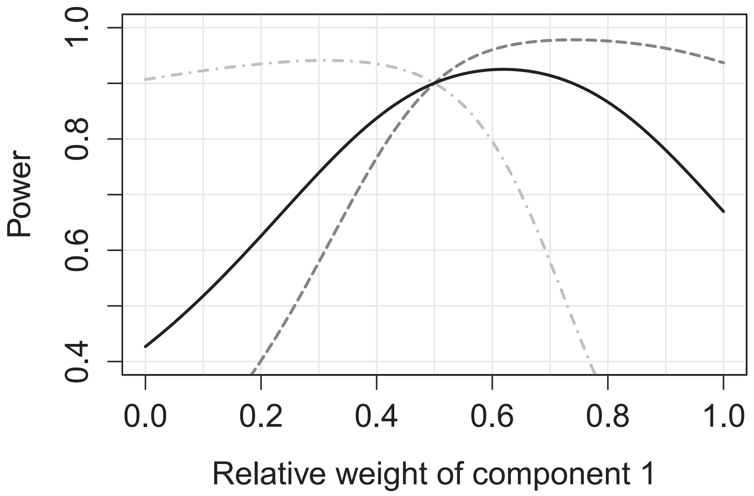

Figure 2. Power of the weighted comparisons of interventions 1 (black solid line), 2 (darkgray dashed line), and 3 (lightgray dash-dotted line) vs. control for a sample size of $n = 503$ subjects per group depending on the weight w_1 assigned to the first component. The weight assigned to the second component w_2 was set to $w_2 = 1 - w_1$.

From Figure 2 it is clear that if specific intervention effects on all component outcomes are assumed, in most situations, a weight combination could be chosen which yielded a higher power than the standard analysis of the composite endpoint (corresponding to equal weights of 0.5). Assuming that component 1 is more clinically relevant, this graph also shows that for intervention 2, which assumes a large effect on component 1, it might be preferable to chose a non-composite outcome which includes only component 1 as the primary endpoint. However, optimizing weights to maximize power is in general not recommended. Rather, as we have stressed, weights should be chosen based on clinical considerations. For example, for intervention 3, putting less weight on component 1 than component 2 leads to a higher power but such weights might not be acceptable from a clinical perspective assuming that component 1 is more severe and clinically relevant.

C. Asymptotic $\bar{\chi}^2$-distributions

We provide a short proof that the exact result of [1] is also valid in the asymptotic setting.

Theorem *Let $\hat{X} \in \mathbb{R}^K$ be a statistic based on a sample of size n, e.g. in Section 3 of the main paper $\hat{X} = \left(\hat{\mathcal{D}}(\tau) - \mathcal{D}(\tau)\right)$, following an asymptotic multivariate normal distribution $\mathcal{N}(0, V)$, where the nonsingular covariance matrix V can be consistently estimated by $n\hat{V}$, i.e.*

$$\sqrt{n}\hat{X} \xrightarrow{D} \mathcal{N}(0, V) \text{ and } n\hat{V} \xrightarrow{P} V$$

where $\xrightarrow{D}$ and $\xrightarrow{P}$ denote convergence in distribution and convergence in probability, respectively. Then for a closed and convex cone $\mathcal{C}$ in $\mathbb{R}^K$,

$$\left[\max_{w\in\mathcal{C}} \frac{\hat{X}^T w}{\left(w^T\hat{V}w\right)^{1/2}}\right]^2 \xrightarrow{D} \bar{\chi}^2.$$

Proof Note that the function $g(y, V, w) = \frac{y^T w}{(w^T V w)^{1/2}}$ is continuous in all of its variables and that

$$\max_{w\in\mathcal{C}} \frac{y^T w}{(w^T V w)^{1/2}} = \max_{w\in\mathcal{C}'} \frac{y^T w}{(w^T V w)^{1/2}}$$

where $\mathcal{C}' = \mathcal{C} \bigcap \left\{w \in \mathbb{R}^k : \|w\| \leq 1\right\}$ is compact. Hence, the function $f(y, V) = \max_{w\in\mathcal{C}} \frac{y^T w}{(w^T V w)^{1/2}}$ is also continuous in (y, V).

According to Theorem 2.7 in [2], $\sqrt{n}\hat{X} \xrightarrow{D} \mathcal{N}(0, V)$ and $n\hat{V} \xrightarrow{P} V$ imply $\left(\sqrt{n}\hat{X}, n\hat{V}\right) \xrightarrow{D} (X, V)$ where X has an exact $\mathcal{N}(0, V)$ distribution. Thus, according to the continuous mapping theorem, we have

$$\left[\max_{w\in\mathcal{C}} \frac{\hat{X}^T w}{\left(w^T\hat{V}w\right)^{1/2}}\right]^2 \xrightarrow{D} \left[\max_{w\in\mathcal{C}} \frac{X^T w}{(w^T V w)^{1/2}}\right]^2$$

and the right-hand side has an exact $\bar{\chi}^2$-distribution according to [1]. $Q.E.D.$

D. Probabilities of the exhaustive event types in the simulation

Tables 2 and 3 below display the observed exhaustive event type proportions in the first and second simulation series, respectively (see Figure 2 of the main paper). These proportions were calculated based on large datasets of size 10^6 simulated according to the respective multi-stage models.

E. R demonstration

In this section we demonstrate how the proposed multiplicity adjustment method can be implemented in the statistical software R [3]. The first part of the code includes general functions for calculating the weights of the relevant $\bar{\chi}^2$-distributions (`wchibarsqa`) and the corresponding cumulative distribution functions (`pchibarsqa`) and quantiles (`qchibarsqa`). This utilizes and extends functions `wchibarsq` and `pchibarsq` from the `R` package `varComp` of

Table 2. Exhaustive event type probabilities with and without right-censoring for the first series.

	$P_k(\tau)$ %		
Group k	N^+F^-	N^-F^+	N^+F^+
No right-censoring before τ			
A_1 & B_1	13.0	8.4	8.1
C_1	11.4	6.6	5.1
D_1	10.1	8.7	6.2
Right-censoring before $\tau^{@}$			
A_1 & B_1	11.8	7.5	7.0
C_1	10.3	5.9	4.3
D_1	9.2	7.7	5.3

Table 3. Exhaustive event type probabilities with and without right-censoring for the second series.

	$P_k(\tau)$ %				
Group k	$N^+M^-F^-$	$N^-M^+F^-$	$N^-M^-F^+$	$N^+M^-F^+$	$N^-M^+F^+$
No right-censoring before τ					
A_2 & B_2	14.0	13.7	12.2	10.2	16.6
C_2	13.6	14.3	10.2	6.8	11.3
D_2	11.0	14.4	12.7	8.0	17.1
Right-censoring before $\tau^{@}$					
A_2 & B_2	13.1	13.1	10.9	8.8	14.4
C_2	12.5	13.2	9.2	5.8	9.8
D_2	10.4	13.7	11.4	6.8	14.8

@: the associated event probabilities display the average observed frequency of those event types, i.e. subjects censored before τ were counted as not having an event for the purpose of this table.

[4] which were written for the special cone $\mathcal{C} = \mathbb{R}_+^K$ to more general cone constraints as defined in equation (4) of the main paper. The second part of the code demonstrates the usage of these functions by reproducing the figure for the enteric trial example discussed in Section 5.2 of the main paper.

```
require(varComp)
require(Matrix)

wchibarsqa <- function (V, R1=NULL, R2=NULL, ...) {
# Weights of the chi-bar-square distribution
# Input:
# - V Covariance matrix of the test statistic
# - R1, R2 The matrices defining the cone by R1 x w >=0 and R2 x w = 0
# - ... Extra arguments for rankMatrix
# Output:
# - wts the weights for the chi-bar-square distribution

 nr1 <- nrow(R1); nc1 <- ncol(R1); nr2 <- nrow(R2); nc2 <- ncol(R2)

 if (is.null(R1) & is.null(R2)) {
  wts <- wchibarsq(solve(V))
 } else if (!is.null(R1) & is.null(R2)) {
 # If the cone is defined by ONLY linear inequality constraints
  if (nr1 == nc1) {
   I1 <- diag(nr1)

   if (max(abs(R1-I1))==0) { # similar to when both R1 & R2 are null
    wts <- wchibarsq(solve(V))
   } else {
    if (det(R1)!=0) {
     wts <- wchibarsq(R1 %*% solve(V) %*% t(R1))
     if(sum(wts)!=1) wts[length(wts)] <- 1-sum(wts[seq_len(length(wts)-1)])
    } else {
     stop("R1 must be nonsingular!")
    }
   }
  } else if (nr1 < nc1) {
   rank1 <- rankMatrix(R1, ...)

   if (rank1 == nr1) {
    wtmp <- wchibarsq(R1 %*% solve(V) %*% t(R1))
    if(sum(wtmp)!=1) wtmp[length(wtmp)] <- 1-sum(wtmp[seq_len(length(wtmp)-1)])
    wts <- rep(0, nrow(V)+1)
    wts[(nc1-nr1+1):(nc1+1)] <- wtmp[1:(nr1+1)]
   } else {
    stop("R1 must be of full row rank")
   }
  } else {
   stop("The number of rows of R1 must not be larger than its number of columns")
  }
 } else if (!is.null(R1) & !is.null(R2)) {
 # If the cone is defined by linear inequality as well as equality constraints
  k <- nr1 + nr2 # total number of rows in R1 and R2

  if (k > nc1 | nc1 != nc2) {
   stop("R1 and R2 must have the same num. of columns and the sum of their row dim must not > their
        common column dim!")
  } else {
   R <- rbind(R1, R2) # equivalently t(cbind(t(R1), t(R2)))
```

```
    raR <- rankMatrix(R, ...)

    if (raR != k) {
      stop("The matrix combined of rows of R1 and R2 must be of full row rank!")
    } else {
      Z <- solve(R %*% solve(V) %*% t(R))[1:nr1, 1:nr1]
      wtmp <- wchibarsq(solve(Z))
      if(sum(wtmp)!=1) wtmp[length(wtmp)] <- 1-sum(wtmp[seq_len(length(wtmp)-1)])
      wts <- rep(0, nrow(V)+1)
      wts[(nc1-k+1):(nc1-nr2+1)] <- wtmp[1:(nr1+1)]
    }
  }
} else if (is.null(R1) & !is.null(R2)) { # Only linear equality
  # The resulting cone is the linear subspace of R^(nc2)
  # The dimension of this subspace is k and rank(R2) + k = nc2
  raR2 <- rankMatrix(R2, ...)
  k <- nc2 - raR2

  if (k == 0) {
    stop("The cone defined by R2 x w = 0 degenerates into a single vector 0!")
  } else {
    wts <- rep(0, nrow(V)+1)
    wts[k+1] <- 1
  }
}

attributes(wts) <- NULL
wts
} # end of wchibarsqa
```

```
pchibarsqa <- function (q, V=NULL, wts=NULL, R1=NULL, R2=NULL, lower.tail = TRUE, log.p = FALSE,
    ...) {
# Cumulative distribution of the chi-bar-square distribution
# ... futher arguments for wchibarsqa or uniroot
 if(is.null(wts)) {
  wts <- wchibarsqa(V, R1, R2, ...)
  if(is.null(wts)) return(NULL)
  n <- nrow(V)
 } else{
  n <- length(wts)-1
 }
 ans <- pchisq(q, 0, lower.tail = FALSE) * wts[1L] +
 pchisq(q, n, lower.tail = FALSE) * wts[n + 1L]
 for (i in seq_len(n - 1)) {
  ans <- ans + pchisq(q, i, lower.tail = FALSE) * wts[i + 1L]
 }
 ans[q <= 0] <- 1
 ans <- if (isTRUE(lower.tail))
 1 - ans
 else ans
 if (isTRUE(log.p))
  log(ans)
 else ans
} # end of pchibarsqa
```

```
qchibarsqa <- function(p, wts=NULL, V=NULL, R1=NULL, R2=NULL, B=2e4, lower.tail=T, ...) {
# Quantiles of the chi-bar-square distribution
# ... futher arguments for wchibarsqa or uniroot
 if (is.null(wts)) {
  wts <- wchibarsqa(V, R1, R2, ...)
  if(is.null(wts)) return(NULL)
  nw <- nrow(V)+1
 } else {
  nw <- length(wts)
 } # end of if (is.null(wts)) else ...

 q <- sapply(p, function(x) {
  upper <- qchisq(x, df=nw-1)
  uniroot(f=function(z) {
   x - pchibarsqa(q=z, wts=wts)
  }, interval=c(0, upper), ...)$root})
 q
} # end of qchibarsq
```

```
covmat.multinom <- function(p){
# Covariance matrix of a multinomial observation, needed for the demonstration below
 cov.p <- -matrix(p,ncol=1)%*%matrix(p,nrow=1)
 diag(cov.p) <- p*(1-p)
 cov.p
} # end of covmat.multinom
```

```
# Typhoid: Cefixime vs. gatifloxacin, see Pandit et al. (2007), figure 1
n.ga <- 92; n.ce <- 77 # total number of subjects in gatifloxacine and cefixime group
n.ga.a <- 1; n.ce.a <- 20 # number of ATF (accute treatment failure)
n.ga.r <- 2; n.ce.r <- 6 # number of relapse

# vector test statistic D and associated covariance matrix cov.D
p.ga <- c(n.ga.a, n.ga.r, n.ga-n.ga.a-n.ga.r) / n.ga
p.ce <- c(n.ce.a, n.ce.r, n.ce-n.ce.a-n.ce.r) / n.ce
ncp <- length(p.ga)
D <- (p.ga - p.ce)[-ncp]
cov.D <- (covmat.multinom(p.ga)/n.ga + covmat.multinom(p.ce)/n.ce)[-(ncp), -(ncp)]

# critical values for chi-bar-square (ad) and unadjusted (un) method
alpha <- .05
calpha.ad <- qchibarsqa(1-alpha/2, V=cov.D, R1=diag(ncp-1))
calpha.un <- qnorm(1-alpha/2)

# weighted risk differences (wds), standard errors (ses) and associated 95% CI
w.as <- seq(0, 1, length.out=1e3) # weight points for ATF
ws <- cbind(w.as, 1-w.as) # weight vectors
ses <- sqrt(apply(ws, 1, function(x) x %*% cov.D %*% x))
wds <- ws %*% D
ci.ads <- cbind(wds - sqrt(calpha.ad) * ses, wds, wds + sqrt(calpha.ad) * ses)
ci.uns <- cbind(wds - calpha.un * ses, wds, wds + calpha.un * ses)
colnames(ci.ads) <- colnames(ci.uns) <- c("low", "est", "upp")

# Nice plot (Figure 3 in the main text)
ci.ads <- data.frame(ci.ads); ci.ads$w.as <- w.as
ci.uns <- data.frame(ci.uns); ci.uns$w.as <- w.as
require("ggplot2");
ggplot(ci.ads) + geom_line(aes(x = w.as, y = low), colour = "grey", alpha = 0.6) +
geom_line(aes(x = w.as, y = upp), colour = "grey", alpha = 0.6) +
geom_line(aes(x = w.as, y = est), colour="black", size=1.5) +
geom_ribbon(aes(x = w.as, ymin = low, ymax = upp), fill = "grey", alpha = 0.6) +
geom_line(data = ci.uns, aes(x = w.as, y = low), colour = "#999999", alpha = 0.4) +
geom_line(data = ci.uns, aes(x = w.as, y = upp), colour = "#999999", alpha = 0.4) +
geom_line(data = ci.uns, aes(x = w.as, y = est), colour="black", size=1.5) +
geom_ribbon(data = ci.uns, aes(x = w.as, ymin = low, ymax = upp), fill = "#999999", alpha = 0.4) +
geom_line(aes(x = w.as, y = est), colour="black", size=1.5) +
guides(size=FALSE, colour=FALSE, fill=FALSE, alpha=FALSE) +
xlab("Rel. weight of acute treatment failure") + ylab("Weighted risk difference") +
theme(axis.text=element_text(size=16), axis.title=element_text(size=18))
```

References

1. Shapiro A. Scheffe's method for constructing simultaneous confidence intervals subject to cone constraints. *Statistics & Probability Letters* 2003; **64**:403–406.
2. Van der Vaart A. *Asymptotic statistics*. 1 edn., Cambridge University Press, 2000.
3. R Core Team. *R: A Language and Environment for Statistical Computing*. R Foundation for Statistical Computing, Vienna, Austria, http://www.R-project.org/ 2014. URL `http://www.R-project.org/`.
4. Qu L. *varComp: Variance component models* 2013. R package version 0.1-317, http://CRAN.R-project.org/package=varComp.
